# Assessment of Health Status, Emotional Well-Being, and the Prevalence of Overweight and Obesity in Children and Adolescents in the PICTURE Study (Wroclaw, Poland)

**DOI:** 10.3390/nu17243817

**Published:** 2025-12-05

**Authors:** Klaudia Konikowska, Krzysztof Kujawa, Agnieszka Matera-Witkiewicz, Katarzyna Połtyn-Zaradna, Katarzyna Zatońska, Tomasz Zatoński, Katarzyna Kiliś-Pstrusińska

**Affiliations:** 1 Department of Dietetics and Bromatology, Faculty of Pharmacy, Wroclaw Medical University, 50-556 Wroclaw, Poland; 2 Statistical Analysis Centre, Wroclaw Medical University, 50-368 Wroclaw, Poland; krzysztof.kujawa@umw.edu.pl; 3 Screening of Biological Activity Assays and Collection of Biological Material Laboratory, Wroclaw Medical University Biobank, Faculty of Pharmacy, Wroclaw Medical University, 50-556 Wroclaw, Poland; agnieszka.matera-witkiewicz@umw.edu.pl; 4 Division of Population Studies and Prevention of Noncommunicable Diseases, Faculty of Health Sciences, Wroclaw Medical University, 50-372 Wroclaw, Poland; katarzyna.poltyn-zaradna@umw.edu.pl (K.P.-Z.); katarzyna.zatonska@umw.edu.pl (K.Z.); 5 Clinical Department of Otolaryngology, Head and Neck Surgery, Faculty of Medicine, Wroclaw Medical University, 50-556 Wroclaw, Poland; tomasz.zatonski@umw.edu.pl; 6 Clinical Department of Paediatric Nephrology, Faculty of Medicine, Wroclaw Medical University, 50-556 Wroclaw, Poland; katarzyna.kilis-pstrusinska@umw.edu.pl

**Keywords:** children, adolescents, health status, emotional problems, overweight, obesity

## Abstract

**Objective**: The aim of the study was to analyze the assessment of the health and emotional well-being of children and adolescents from Wroclaw, including the frequency of diseases, health symptoms, overweight, and obesity. **Methods**: The study was conducted as a cross-sectional study between 2019–2023, ultimately involving 1232 children aged 7–17 years. The data were collected in the form of interviews with caregivers. The study used a health questionnaire, anthropometric measurements and laboratory tests. Overweight and obesity were determined based on Polish body mass index (BMI) percentile tables in accordance with pediatric society guidelines. For the purposes of analysis, participants were divided into three age groups: 6–9, 10–12, and 13–17 years old. **Results**: The most common diagnosis was allergies, affecting about 36% of those surveyed. In total, 32.8% of children reported difficulties with concentration, memory, and learning. Emotionally, over one-third of children experienced anxiety or fear and outbursts of anger, 26% experienced inadequate sadness, and about 22% complained of chronic fatigue and excessive agitation. About 15% of participants reported symptoms of depression. Overweight and obesity were present in about 18% of children, with the frequency increasing with age. Moreover, it was shown that gender and age were the most important factors differentiating the risk of emotional symptoms, while body weight had no significant effect on any of the analyzed symptoms. **Conclusions**: The study revealed a significant prevalence of emotional disorders among children and adolescents, an increasing prevalence of allergies with age, and the growing problem of overweight and obesity. The results indicate the need for the implementation of systematic preventive measures and the early diagnosis of chronic diseases. An effective response to these challenges requires the development of an interdisciplinary healthcare model that integrates pediatric, psychologist, dietary, and social support.

## 1. Introduction

Childhood and adolescence are crucial stages of life during which numerous habits are formed that influence both current health status and future health outcomes [1]. The deterioration of health and disruptions in normal development among children and adolescents are influenced by factors such as chronic illnesses, emotional disorders, and problems related to excessive body weight. The health status of children and adolescents worldwide represents a significant area of research.

The presence of chronic diseases in children and adolescents can adversely affect their quality of life [2]. Long-term illness can have a negative impact on a student’s life, reducing school attendance and engagement in learning, and significantly hindering their developmental potential [2,3]. Meanwhile, mental health, particularly among adolescents, has only recently become a focus of researchers [4]. Azzopardi et al. [4] observed that the decline in adolescent health and well-being outcomes coincided with the COVID-19 pandemic, which had a distinctly negative impact on public health, especially among adolescents. Experts from the World Health Organization (WHO) emphasize that the health and well-being of children and adolescents are key to achieving the Sustainable Development Goals (SDGs) [5].

One of the threats to the health of young people is excessive body weight, which negatively impacts their overall health. Overweight and obesity represent an increasingly daunting challenge. According to WHO estimates, one in three school-aged children is affected by excessive body weight [6]. Existing research indicates a rising trend of overweight and obesity among Polish children [7]. Overweight and obesity are major risk factors for non-communicable diseases, including cancers, type 2 diabetes, and cardiovascular diseases [6,8]. The causes of excessive body weight in children and adolescents include a positive energy balance, unhealthy eating habits (e.g., insufficient intake of fruits and vegetables, excessive consumption of high-calorie snacks and sugary beverages), insufficient or lack of physical activity, prolonged screen time, and inadequate sleep hygiene [9]. Another factor that exacerbated the issue of excessive body weight in both children and adolescents was the COVID-19 pandemic [6,9,10].

In response to these challenges, the PICTURE project (Population Cohort Study of Wroclaw Citizens) was launched to assess the health status and lifestyle of school-aged children and their caregivers. The PICTURE project is part of the long-term public health strategy of the Wroclaw metropolitan area [11]. Additionally, the study serves as an effective tool for identifying biopsychosocial factors that differentiate health risks within the studied population. As part of the project, a cohort of participants was created and is subject to long-term observation, with follow-up assessments conducted every two years [11]. Due to the wide range of analyzed factors, PICTURE is among the most comprehensive cohort studies involving children in Poland [11]. This study fills a research gap in the comprehensive assessment of the health status of Polish children and adolescents. The findings of the PICTURE study will support the implementation of new educational, preventive, and intervention programs aimed at improving health, with particular emphasis on the younger generation.

The aim of the study was to assess the health status and emotional well-being of children and adolescents participating in the PICTURE project, with particular emphasis on the frequency of diseases, health symptoms, overweight and obesity.

## 2. Materials and Methods

The present study utilized baseline data from the PICTURE program, a cohort study involving school-aged children and their caregivers. Initially, the first stage of recruitment for the PICTURE study involved a group of 3750 children aged 7–14, randomly selected from the PESEL database and stratified by age and sex, all of whom were registered residents of the city of Wroclaw in 2019 (inclusion criteria). In the second stage of recruitment in 2021—1250 children were also randomly selected from the PESEL database. A total of 5000 children and adolescents were randomly selected. Invitations to participate in the study were sent by letter. Ultimately, 1232 children and adolescents aged 7 to 17 participated in the study. Data from children, adolescents, and their caregivers were collected between 2019 and 2023. This study is the first in a series focusing on the pediatric population, provides a comprehensive overview of the health status of children and adolescents in Wroclaw, Poland. It presents data on the prevalence of diseases and clinical symptoms, overweight and obesity, and emotional and behavioral problems. These results provide valuable baseline for future analyses that will allow for a more detailed examination of dietary habits, physical activity, sleep, and screen time in this cohort.

The project was designed to conduct longitudinal analysis, forming a Wroclaw-based cohort. Data was collected in a standardized manner, at two-year intervals, with annual follow-up via telephone contact [11]. Health information regarding the children was obtained through anamnesis with parents or legal guardians, grandparents, or a parent’s partner. The detailed methodology of the PICTURE study is described in the research protocol published by Zatońska et al. [11]. In this analysis, a cross-sectional study design was applied.

This publication characterizes the health status of children and adolescents in Wroclaw aged 7–17 years. For analytical purposes, study participants were divided into three age groups: 6–9 years (Group A), 10–12 years (Group B), and 13–17 years (Group C). The analysis included data on health problems, reported diseases, and emotional states within the studied group. These data were collected using a child health questionnaire comprising 203 parameters, such as: assessment of health status in the opinion of the caregiver, diagnosed diseases and their course, pharmacological treatment, basic information about emotional state, diet, and eating habits [11]. Laboratory test results from the examined children were also compiled. Each participant underwent the following laboratory tests: complete blood count, blood glucose concentration, lipid profile (total cholesterol, LDL, HDL, triglycerides (TGs), and calculated non-HDL), creatinine, TSH, electrolytes (sodium and potassium), and HbA1c). Anthropometric measurements, including height and body weight, were taken for every child. Based on these values, body mass index (BMI) was calculated, and growth, weight, and BMI percentiles were determined using the OLAF calculator [12]. The calculations in the OLAF calculator were prepared based on the work of Kułaga et al. [13]. The prevalence of overweight and obesity in the PICTURE study was determined according to Polish BMI percentile charts. Overweight was defined as BMI > 85th percentile (>1 SD), and obesity as BMI > 97th percentile (>2 SD), following the recommendation of the Polish Pediatric Society, the Polish Society for Pediatric Obesity, the Polish Society of Pediatric Endocrinology and Diabetology, the College of Family Physicians in Poland, and the Polish Association for the Study of Obesity regarding childhood obesity [9]. A BMI value below the 5th percentile was classified as underweight.

The primary outcomes in the manuscript were indicators of the incidence of emotional disorders, and overweight and obesity. Secondary outcomes included other aspects of health functioning, including the occurrence of health symptoms in the past year, occurrence of chronic diseases, laboratory test results, and information regarding the use of psychological or psychiatric services. The study defined measurable endpoints that formed the basis for assessing the health and well-being of participants. These included the occurrence of disease symptoms in the last year (answers “yes” or “no”), chronic diseases (answers “yes” or “no” with details of the disease), emotional disorders (answers “yes” or “no” for specific emotional conditions). The endpoints also included: use of the support of a psychologist or psychiatrist (answers “yes” or “no”), occurrence of overweight or obesity determined on the basis of BMI (in relation to age and gender norms), and laboratory test results compared with reference values.

Prior to participation, written informed consent was obtained from each child’s parent or legal guardian, as well as from the child if aged 13 or older, for both participation in the study and the biobanking of biological material. By signing the informed consent form, participants were made aware that collected samples and related data would not be shared with third parties for purposes other than scientific research. The materials and data are made available for scientific purposes only in anonymized form. Surveys and medical anamneses were conducted with full respect for participant privacy, personal data protection, and the comfort and safety of the child.

The PICTURE study was conducted in accordance with the Declaration of Helsinki and approved by the Bioethics Committee of the Wroclaw Medical University (Opinion No KB-667/2019) on 7 October 2019.

### Statistical Analysis

Quantitative parameters in all subgroups were tested for normality using the Kolmogorov–Smirnov test or the Shapiro–Wilk test (for subgroup sizes below 50), with a significance level of *p* < 0.05. As the distribution of many variables significantly deviated from normality, the analyzed variables were described using medians and quartiles, and intergroup differences were evaluated using the non-parametric Kruskal–Wallis test with Dunn’s post hoc test (with Bonferroni correction). The η^2^ (eta squared) was used as the effect size measure for the Kruskal–Wallis test. Nominal characteristics of the study group were presented in tables as counts (n) and percentages (%). Hypotheses regarding the independence of nominal and ordinal features were verified using Pearson’s Chi-squared test. The Cramer’s V was used as the effects size measure for this test. For all statistical tests, a significance level of *p* < 0.05 was adopted. The analyses were done using original, non-transformed data. No missing data has been imputated.

Multivariate analysis using logistic regression was conducted to assess the relationship between the occurrence of individual emotional disorders (anxiety or fear, inadequate sadness, depression, excessive agitation, chronic fatigue, anger attacks) and potential predictors: gender, age, excess body weight (overweight and obese combined) and underweight. Each variable from the individual emotional disorders was analyzed as an explained variable in a separate model. Each model included all predictors simultaneously (full model). Odds ratios (ORs) were calculated for each factor, along with 95% confidence intervals (CI) and *p*-values. The discriminatory power of the models was assessed using the C-index (corresponding to the area under the ROC curve). Model fit was additionally assessed using the Nagelkerke R^2^ coefficient. Logistic regression assumptions were checked using the Box-Tidwell test (for the linearity of the logit with continuous predictor, i.e., age), and the Generalized Variance Inflation Factor (for the absence of multicollinearity).

Statistical analysis was performed using the Statistica v.13 software package (StatSoft Inc., Tulsa, OK, USA) and R-packages for calculation of η^2^ (‘rstatic’ 0.7.2), Nagelkerke R^2^ (‘rcompanion’ 2.4.36), C-index (‘DeskTool’ v. 0.99.58), and VIF (‘rms’ v. 6.8-2) which was run in R 4.5.1.

## 3. Results

The study included 1232 children aged 7 to 17 years (Me = 12.0; Q1–Q3 = 10.0–13.0). Among them, there were 611 girls (49.6%) and 621 boys (50.4%).

### 3.1. Health Status of Children as Assessed by Their Caregivers

In the conducted study, caregivers responded to questions concerning the occurrence of various disease symptoms in their children over the past year. The symptoms reported and responses obtained are presented in Table 1. The results indicated that the most frequently reported symptoms among children and adolescents were headaches (38.7%) and gastrointestinal symptoms such as abdominal pain, digestive problems, constipation, or diarrhea (38.1%). A slightly lower proportion of respondents reported symptoms related to dental health. Dental caries was noted in 31.7% of the participants. Attention, memory, and learning difficulties were reported at a similar frequency (32.8%). A less common but notable symptom was vision impairment or visual difficulties, reported in 12.8% of the cohort. Issues such as nocturnal enuresis (1.7%) and daytime urinary incontinence (1.4%) were rare.

Among the youngest children (aged 7–9), dental caries in the past year was the most frequently reported condition (45.8%). Conversely, headaches, gastrointestinal symptoms, and vision problems were most commonly observed in the oldest age group (13–17 years) at rates of 48.8%, 43.5%, and 18.2%, respectively. Attention, memory, and learning difficulties were similarly prevalent in the 10–12 and 13–17 age groups (34.1% and 35.6%, respectively).

### 3.2. Physician-Diagnosed Diseases in Children

In the section of the questionnaire related to chronic diseases, caregivers were asked about specific conditions diagnosed by a physician. The most frequently diagnosed condition was allergies, affecting over 35% of the study population. The highest prevalence was found in the oldest age group (13–17 years) at 41.3%. Other conditions were diagnosed considerably less frequently. About 3–4% of children had musculoskeletal disorders, thyroid disease, heart disease, asthma, and gastrointestinal disorders. Musculoskeletal and thyroid disorders were most frequently diagnosed in the oldest age group (6.6% and 5.3%, respectively) and least frequently in the middle age group (10–12 years) at 0.4% each. Detailed data on the occurrence of diseases in the entire cohort and across age groups are presented in Table 2.

### 3.3. Laboratory Test Results

As part of the PICTURE study, a range of laboratory tests were conducted among children and adolescents, including complete blood counts, total cholesterol and its fractions, fasting glucose, glycated hemoglobin, and thyroid hormones. Key laboratory findings are summarized in Table 3. Significantly higher hemoglobin levels were observed in the 13–17 age group compared to younger children (*p* < 0.001). Additionally, this group exhibited significantly lower levels of total cholesterol, non-HDL-C, and LDL-C compared to children aged 7–9 and 10–12 years (*p* < 0.001). TG levels varied significantly between age groups (*p* < 0.001). TG levels were significantly lower in the youngest group (7–9 years) compared to both the middle (10–12 years) and oldest (13–17 years) groups. Significant differences in TG levels were also noted between the middle and oldest age groups, with lower levels in the former. The highest HDL cholesterol levels were recorded in the youngest children. The lowest fasting glucose concentrations were also observed in the youngest group compared to the two older groups (*p* < 0.001). Significant differences in TSH and glycated hemoglobin levels were identified between the 10–12 and 13–17 age groups (*p* = 0.006 and *p* = 0.003, respectively).

### 3.4. Emotional Disorders in the Study Population

**Figure 1:**
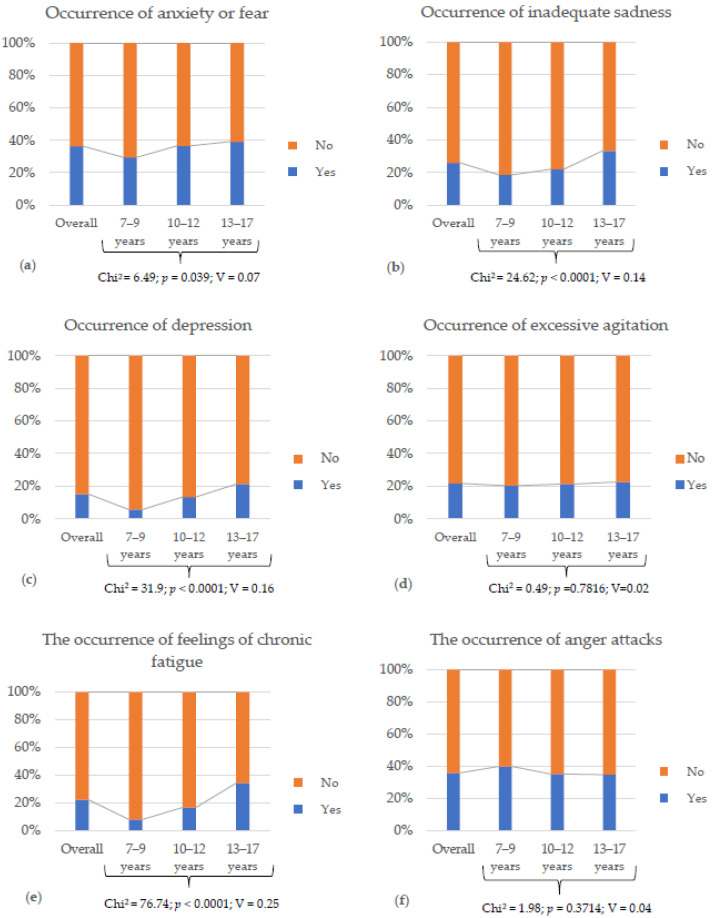
The frequency of emotional disorders in the last year in the entire study group of children and adolescents and in individual age groups (7–9 years, 10–12 years, 13–17 years): (**a**) Occurrence of anxiety or fear; (**b**) Occurance of inadequate sadness; (**c**) Occurrence of depression or depressed mood state; (**d**) Occurrence of excessive agitation; (**e**) Occurrence of feelings of chronic fatigue; (**f**) Occurrence of anger attacks. Chi^2^—test value; *p* value—statistical significance; V—Cramer’s coefficient.

Over one-third of the participants experienced anxiety or fear and anger outbursts in the 12 months preceding the study (36.3% and 36%, respectively). Feelings of inappropriate sadness were reported by 25.9% of the children. Approximately 22% experienced chronic fatigue, and a similar proportion (21.6%) reported hyperactivity. Depression or a depressive mood was reported in about 15% of the children. Statistically significant differences were observed between age groups in the prevalence of anxiety, inappropriate sadness, depression, and chronic fatigue. Notably, over 39% of adolescents aged 13–17 experienced anxiety. The lowest incidence of anxiety was recorded in the youngest group (approximately 29%). An inverse pattern was observed for anger outbursts, though no statistical difference between groups was found. The youngest children (7–9 years) were most prone to anger outbursts (39.9%), while this symptom was less common among the oldest (34.8%) and middle (35.2%) age groups. In the oldest group, inappropriate sadness (33.3%), chronic fatigue (34.3%), and depressive symptoms (21.2%) were most prevalent. These emotional disorders were least frequently observed in the youngest children. Detailed results concerning emotional disturbances over the past year for the entire study population and by age group are presented in Figure 1.

**Figure 2:**
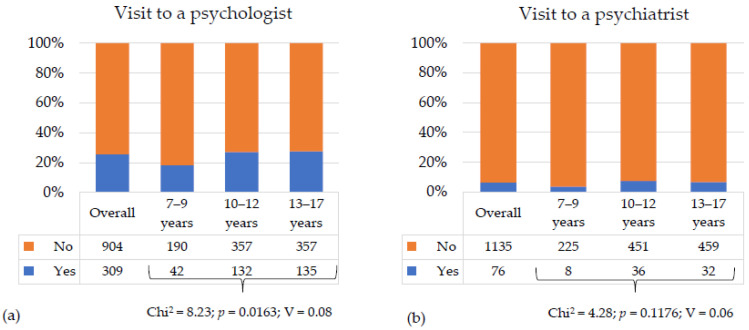
Frequency of visits to a psychologist and psychiatrist in the last year among all children and adolescents and by age group (7–9 years, 10–12 years, 13–17 years): (**a**) Visit to a psychologist; (**b**) Visit to a psychiatrist. Chi^2^—test value; *p* value—statistical significance; V—Cramer’s coefficient.

Approximately 25% of children had visited a psychologist in the year before the study. The highest proportions were observed in the middle (10–12 years) and oldest (13–17 years) age groups, at 27% and 27.4%, respectively. The lowest proportion of psychological visits occurred in the youngest group (7–9 years), at approximately 18%. Around 6% of children had visited a psychiatrist in the year before the study, with no significant differences between age groups. Detailed data on visits to psychologists and psychiatrists are presented in Figure 2.

To identify factors associated with the occurrence of the studied emotional disorders, both univariate analysis and multivariate logistic regression were conducted. In the first stage, the relationships between predictors and emotional symptoms were assessed in an unadjusted manner, while multivariate models simultaneously considered gender, age, excess body weight (as overweight and obese together), and underweight. The model results are presented in Table 4. The interpretation of the results is presented below, focusing on statistically significant relationships.

In a model that considered all predictors simultaneously, gender was found to be a significant factor associated with anxiety or fear (*p* = 0.001). Boys had a 32% lower odds of anxiety or fear compared to girls (OR = 0.680; 95% CI: 0.537–0.862). Age showed a borderline significant relationship (OR = 1.049; *p* = 0.062), suggesting a possible, though ambiguous, trend toward increased risk with age. In next model, boys had approximately 53% lower odds of experiencing inappropriate sadness than girls (OR = 0.471; CI: 0.361–0.616; *p* < 0.001). However, each additional year of age increased the risk of inappropriate sadness by 12% (OR = 1.120; OR: 1.060–1.184; *p* < 0.001). The third model found that boys had approximately a 57% lower odds of experiencing depressive symptoms than girls (OR = 0.428; CI: 0.305–0.601; *p* < 0.001). However, with age, the odds of depression increase by 23% annually (OR = 1.231; OR: 1.147–1.320; *p* < 0.001). In the fourth model, statistical significance was observed only for gender. Boys had approximately 48% higher odds of excessive agitation than girls (OR = 1.484; CI: 1.124–1.959; *p* = 0.005). In the next model, which considered all predictors simultaneously, both gender and age were found to be significantly associated with the occurrence of chronic fatigue (*p* = 0.010; *p* < 0.001). Boys had approximately 31% lower odds of experiencing chronic fatigue than girls (OR = 0.688; CI: 0.518–0.913; *p* = 0.010). Each year of age increased the odds of experiencing chronic fatigue by 32% (OR = 1.320; CI: 1.240–1.406; *p* < 0.001). In the final model, only gender was found to be a significant factor associated with the occurrence of anger attacks (*p* = 0.046). It was shown that boys had approximately 27% higher odds of anger attacks than girls (OR = 1.272; CI: 1.004–1.612; *p* = 0.046). Excessive body weight and underweight were not significantly associated with the occurrence of any of the above emotional disorders in these models.

It should be emphasized that all models had poor discriminatory power. The best-fitting model was Model III (depression). The models also had low explanatory power (low Nagelkerke R^2^). The best-fitting model was Model V (chronic fatigue), explaining only 11.7% of the variance.

### 3.5. Prevalence of Overweight and Obesity

**Figure 3:**
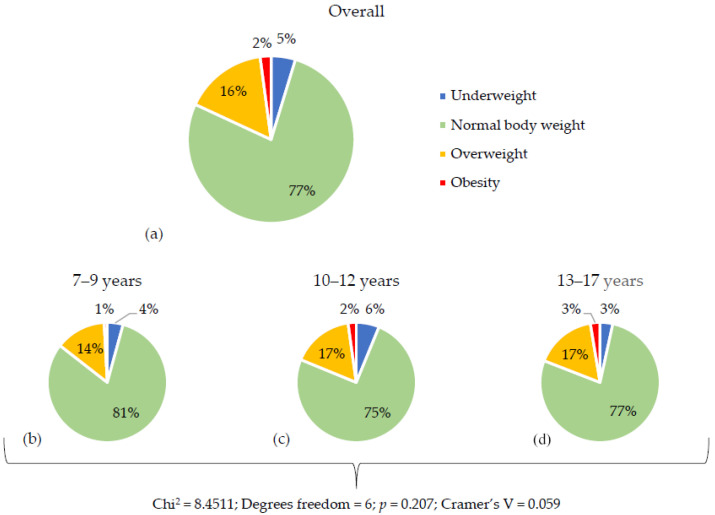
The prevalence of underweight, normal body weight, overweight and obesity in the entire study group of children and adolescents and by age group (7–9 years; 10–12 years; 13–17 years): (**a**) Body weight profile in the entire study group; (**b**) Body weight profile of children aged 7–9 years; (**c**) Body weight profile of children aged 10–12 years; (**d**) Body weight profile of children aged 13–17 years. Chi^2^—test value; *p* value—statistical significance; V—Cramer’s coefficient.

BMI classification, including normal weight, overweight, obesity, and underweight, is presented in Figure 3. Normal BMI was observed in 951 children (approximately 77%) participating in the PICTURE study. Overweight was found in 197 children (16%), obesity in 26 (approximately 2%), and underweight in 58 (approximately 5%). In the youngest group, overweight was diagnosed in 32 children (approximately 14%) and obesity in 2 (approximately 1%). In the middle age group, overweight and obesity were found in 83 (approximately 17%) and 11 (approximately 2%) children, respectively. In the oldest group, 82 (approximately 17%) were overweight and 13 (approximately 3%) were obese.

## 4. Discussion

The PICTURE study provides valuable insights into the health of children and adolescents residing in Wroclaw, Poland. The analysis of disease symptoms revealed that the most common complaints reported by caregivers were headaches and gastrointestinal issues, possibly highlighting the role of factors such as diet, stress, or sleep hygiene. Notably, dental caries was most frequent in the youngest group, suggesting a need for intensified oral hygiene preventive measures.

The analysis of diagnosed conditions revealed that allergies were the most prevalent, particularly in adolescents, reflecting a rising trend during puberty. This may be associated with environmental pollution and changes in immune function. Urrutia-Pereira et al. [14] documented that prenatal and postnatal exposure to air pollution increases the risk of illness and mortality from numerous conditions, including allergies. Air pollution disrupts the microbiome, alters immune responses, and directly affects the skin and respiratory epithelium, facilitating allergen penetration [14].

Other conditions, such as musculoskeletal, thyroid, or cardiovascular disorders, occurred less frequently but were more commonly diagnosed in older children, potentially indicating an age-related progression of health issues.

Laboratory findings offered crucial information regarding children’s metabolic health. The oldest group had significantly higher hemoglobin levels, possibly reflecting the physiological demands of puberty. However, this group also showed lower mean levels of total cholesterol, LDL, and non-HDL cholesterol. Of concern, TG levels increased significantly with age, likely reflecting dietary and physical activity changes. There is substantial evidence that atherosclerotic plaque formation begins in childhood and adolescence, possibly leading to early cardiovascular complications [15]. According to the National Health and Nutrition Examination Survey (NHANES), 20% of adolescents aged 12–19 have lipid disorders [16], which often remain undiagnosed due to their asymptomatic nature [15]. Early identification and appropriate treatment of dyslipidemia in children may significantly reduce future cardiovascular risk.

The results of laboratory tests allowed for an assessment of the overall health of the children and adolescents examined. However, mental health also remains an important element of well-being. Emotional disorders were a substantial issue in the study population. Over one-third of children experienced anxiety or fear, with adolescents being the most affected. Conversely, anger outbursts were most frequent among the youngest children. These findings underscore the need for age-appropriate psychological support. Nearly one in four children visited a psychologist in the previous year, highlighting the increasing demand for mental health services. Anxiety disorders are the most commonly diagnosed mental illnesses among children and adolescents, and untreated anxiety often persists into adulthood [17]. Moreover, adolescents with such disorders are more susceptible to developing additional mental health issues, such as depression, substance abuse, or early school dropout [18]. The COVID-19 pandemic has had a substantial negative impact on youth mental health worldwide [19,20]. In a review by Samji et al. [21], 25 articles reported a higher prevalence of depressive symptoms in children and adolescents during the pandemic compared to the pre-pandemic period. Most studies measured anxiety symptoms in young people, and 17 of them found elevated levels of anxiety symptoms compared to pre-pandemic estimates [21].

Both in Poland and globally, limited access to psychological and psychiatric care remains a challenge, leading to delays in treatment initiation [19,22,23,24]. Optimal solutions for improving access to mental health care are still lacking [19]. It is also worth noting the need for systematic monitoring of the mental health of children and adolescents and strengthening their mental resilience [25]. Mesman et al. [25] noted in all studies included in their review that higher levels of mental resilience were associated with fewer mental health problems in children and adolescents. They also showed that poorer quality interpersonal relationships with parents, teachers, or peers [26,27,28]. The study Lee et al. [26] showed that better interpersonal relationships were associated with higher levels of mental resilience and fewer symptoms of depression.

The well-being of children and adolescents is influenced not only by emotional factors, but also by physical factors, including body weight. BMI analysis showed that most children had a normal body weight, but about 18% were overweight or obese. The prevalence of excess weight increased with age. In the international Health Behaviour in School-aged Children (HBSC) study, the prevalence of overweight and obesity in Polish adolescents (based on IOTF criteria) was 14.8% in 2014, rising to 16.5% in 2018 [29,30]. Miguel-Berges et al. [31] found that approximately 26% of Spanish children were overweight, with 18% classified as overweight and 8% as obese. Vasiljevic and Petkovic [32] reported a 28% prevalence of overweight and obesity among Montenegrin children aged 6–9 years (15% overweight, 13% obese). Gioxari et al. [33] found that around 35% of Greek children aged 6–11 were overweight or obese based on z-BMI. A systematic review by Santos et al. [34] showed that approximately 12% of Brazilian children were obese. Overweight and obesity are among the fastest-growing health issues in children and adolescents, contributing to physical and emotional complications in adulthood and posing significant economic burdens on healthcare systems and society [1]. Implementing healthy dietary habits and physical activity can significantly improve children’s and adolescents’ health and should be a key component in addressing excess weight [1]. Tackling overweight and obesity is a global challenge, including in European countries, and requires both health education and continuous weight monitoring among youth.

Although the study mainly focuses on descriptive characteristics, it provides unique data on the physical, mental and metabolic health of children and adolescents from Wroclaw. The integration of these aspects is rarely presented in Polish population studies, which allows for a more holistic view of the well-being of young people.

The observed patterns indicate the need for targeted preventive measures. The results of this study emphasize the need for: early identification of emotional disorders in schools, improved access to psychological and psychiatric care, mandatory health education for all pediatric age groups, consideration of regular lipid screening for adolescents, and strengthening dental preventive programs for the youngest children.

The results also reveal areas requiring further cohort follow-up, such as the relationship between mental health and health behaviors, metabolic changes during adolescence, and the impact of the environment and stress on children’s physical health.

### Limitations and Strengths of the Study

The study has certain limitations. Information regarding the occurrence of disease symptoms and emotional states in children was collected using questionnaires completed by the caregivers of the participating children. These data may be subject to recall bias and reporting bias. These biases may result from memory limitations, subjective interpretation of the situation (proxy perception bias), and a tendency to present information in a socially acceptable manner (social desirability bias). In this type of research, there is always a risk that caregivers may overreport or underreport the data. The interpretation of the obtained results should be conducted with caution. The study’s participation rate was less than 30%, which could be a potential source of selection bias. Participants who consented to participate may differ from those who declined on important variables. For this reason, caution was exercised in interpreting the results and generalizing the conclusions to the general population.

However, the study has several strengths. The questionnaires applied in our study were based on tools previously used by the authors in the PURE (Prospective Urban and Rural Epidemiological Study) [35,36], as well as in the “Let’s get the kids moving” project [11,37]. This pertains to questionnaires related to the health status and lifestyle of the participants. Another strength lies in the recruitment method used for the PICTURE study, which involved a randomized selection of participants from the PESEL database, stratified by age and sex.

## 5. Conclusions

Our research results indicate a significant prevalence of emotional disorders among children and adolescents. In the context of recognizing the mental health of children and young people as a priority of the Polish Presidency, it is necessary to strengthen systemic actions. These should be focused on early identification of emotional problems. It is advisable to increase access to psychological and psychiatric support, especially in the school environment, by ensuring an adequate number of specialists, developing preventive programmes and integrating activities between schools, counselling centres and local government units.

In terms of disease prevalence, allergies were the most commonly diagnosed conditions, with their frequency increasing with age. Similarly, musculoskeletal and thyroid disorders were more prevalent among adolescents, which may suggest a growing burden of health issues during puberty. These findings indicate the need for regular screening among children and adolescents and for strengthening educational efforts for the early detection of chronic diseases. In public health practice, it is advisable to implement school-based programs on chronic disease prevention and health education for parents and teachers.

The health status of Polish children in terms of overweight and obesity is comparable to the situation observed in other European countries. However, the observed increase in excess body weight with age is a significant public health problem that requires integrated systematic interventions. It is advisable to create and develop specialized centers for the comprehensive treatment of overweight and obesity in children. These centers should operate based on an interdisciplinary care model, including the cooperation of a pediatrician, dietitian, psychologist, psychiatrist, physiotherapist, and public health specialist. The popularization of such centers in Poland, along with the formal recognition of the dietitian profession as a medical profession, would be a significant step towards strengthening the effectiveness of preventive and therapeutic activities and improving the quality of care for children and adolescents with excess body weight.

The results of multivariate analyses revealed that gender and age were key predictors of selected emotional disorders, while body weight was not significantly associated with any of the assessed symptoms. These results emphasize the multifactorial aspect of emotional disorders and indicate the need to consider a broader psychological and environmental context in further research,

In summary, the results of the PICTURE study have provided valuable insights into the health status of children and adolescents and point to significant health challenges within this population. The findings highlight the need for coordinated preventive and intervention efforts in three key areas: mental health, chronic disease, and obesity. Regular monitoring of children and adolescents’ health, combined with improved access to interdisciplinary care, can contribute to improving their well-being and quality of life.

## Figures and Tables

**Table 1:** Occurrence of individual disease symptoms over the past year in a group of children based on caregivers’ opinions.

Variables		Overall n (%)	Age Group			Chi^2^	*p*-Value	V
			7–9 Years n (%)	10–12 Years n (%)	13–17 Years n (%)			
Caries (n = 1211)		384 (31.7%) 827 (68.3%)	136 (45.8%) 161 (54.2%)	163 (32.9%) 332 (67.1%)	85 (20.3%) 334 (79.7%)	52.8	<0.001	0.21
	Yes							
	No							
Abdominal pain, digestive problems, constipation or diarrhea (n = 1213)		462 (38.1%) 751 (61.9%)	103 (34.6%) 195 (65.4%)	176 (35.6%) 318 (64.4%)	183 (43.5%) 238 (56.5%)	8.0	0.0182	0.08
	Yes No							
Headaches (n = 1208)		468 (38.7%) 740 (61.3%)	71 (24.0%) 225 (76.0%)	192 (39.0%) 300 (61.0%)	205(48.8%) 215(51.2%)	45.1	<0.001	0.19
	Yes No							
Problems with focusing attention, remembering, learning (n = 1212)		397 (32.8%) 815 (67.2%)	79 (26.5%) 219 (73.5%)	168 (34.1%) 325 (65.9%)	150 (35.6%) 271 (64.4%)	7.2	0.0267	0.08
	Yes No							
Visual disorders/vision problems (n = 1205)		154 (12.8%) 1051 (87.2%)	19 (6.4%) 278 (93.6%)	59 (12.0%) 431 (88.0%)	76 (18.2%) 342 (81.8%)	22.0	<0.001	0.14
	Yes No							
Bedwetting (n = 1204)		21 (1.7%) 1183 (98.3%)	12 (4.1%) 282 (95.9%)	6 (1.2%) 486 (98.8%)	3 (0.7%) 415 (99.3%)	12.7	0.0017	0.10
	Yes No							
Daytime urinary (n = 1205)		17(1.4%) 1188 (98.6%)	4 (1.3%) 293 (98.7%)	7 (1.4%) 485 (98.6%)	6 (1.4%) 410 (98.6%)	0.0	0.994	0.00
	Yes No							

Chi^2^—test value, V—Cramer’s coefficient.

**Table 2:** Prevalence of specific diseases in a group of children based on survey responses from caregivers.

Variables	Overall n (%)	Age Group			Chi^2^	*p* Value	V
		7–9 Years n (%)	10–12 Years n (%)	13–17 Years n (%)			
Allergies Yes No	431 (35.7%) 776 (64.3%)	149 (30.7%) 337 (69.3%)	79 (34.3%) 151 (65.7%)	203 (41.3%) 288 (58.7%)	12.4	0.0021	0.10
Asthma Yes No	43 (3.6%) 1158 (96.4%)	23 (4.8%) 461 (95.2%)	7 (3%) 224 (97%)	13 (2.7%) 473 (97.3%)	3.3	0.1938	0.05
Heart diseases Yes No	39 (3.3%) 1160 (96.7%)	10 (2.1%) 474 (97.9%)	9 (3.9%) 223 (96.1%)	20 (4.1%) 463 (95.9%)	3.7	0.1600	0.06
Thyroid diseases Yes No	43 (3.6%) 1161 (96.4%)	16 (3.3%) 471 (96.7%)	1 (0.4%) 230 (99.6%)	26 (5.3%) 460 (94.7%)	11.2	0.0037	0.10
Diseases of the osteoarticular system Yes No	47 (3.9%) 1156 (96.1%)	14 (2.9%) 473 (97.1%)	1 (0.4%) 230 (99.6%)	32 (6.6%) 453 (93.4%)	18.2	0.0001	0.12
Diseases of the digestive system Yes No	40 (3.3%) 1159 (96.7%)	18 (3.7%) 465 (96.3%)	7 (3%) 225 (97%)	15 (3.1%) 469 (96.9%)	0.4	0.8245	0.02

Chi^2^—test value, V—Cramer’s coefficient.

**Table 3:** Results of laboratory tests in the group of studied children.

Variables	Age Group			N	H	η^2^	*p* Value
	7–9 Years (A) Me (Q1, Q3)	10–12 Years (B) Me (Q1, Q3)	13–17 Years (C) Me (Q1, Q3)				
Hemoglobin [g/dL]	13.2 (12.7, 13.7)	13.4 (12.9, 13.8)	13.7 (13.1, 14.4)	1223	68.78	0.054	<0.001 * (A vs. C <0.001; B vs. C <0.001)
Total cholesterol [mg/dL]	162 (147.5, 182)	163 (149, 183)	151 (135, 168)	1218	78.20	0.062	<0.001 * (A vs. C <0.001; B vs. C <0.001)
HDL cholesterol [mg/dL]	63.3 (55.8, 71.6)	61.7 (53.2, 70)	55.7 (48.2, 63.9)	1219	77.19	0.061	<0.001 * (A vs. C <0.001; B vs. C <0.001)
non-HDL cholesterol [mg/dL]	98.1 (83.8, 119)	99.7 (84.8, 119.6)	92.7 (78.5, 110.5)	1219	28.46	0.021	<0.001 * (A vs. C = 0.004; B vs. C <0.001)
LDL cholesterol [mg/dL]	86.2 (73, 103.6)	85.6 (72.3, 104.1)	78.1 (63.4, 93.6)	1219	41.98	0.032	<0.001 * (A vs. C <0.001; B vs. C <0.001)
TGs [mg/dL]	57 (42, 71)	63 (48, 88)	68 (53, 90)	1219	39.80	0.031	<0.001 * (A vs. B <0.001; A vs. C <0.001; B vs. C = 0.019)
FG [mg/dL]	84 (79, 88)	85 (81, 89)	86 (82, 90)	1221	22.54	0.017	<0.001 * (A vs. B = 0.012; A vs. C <0.001)
TSH [mlU/L]	2.2 (1.6, 3.0)	2.2 (1.7, 2.8)	2 (1.5, 2.8)	1217	11.02	0.007	0.0040 * (B vs. C = 0.006)
HbA1C [%]	5.3 (5.1, 5.4)	5.3 (5.1, 5.4)	5.2 (5.1, 5.4)	1201	11.64	0.008	0.0030 * (B vs. C = 0.003)

HDL—high-density lipoprotein; LDL—low-density lipoprotein; TGs—triglycerides; FG—fasting glucose; TSH—thyroid-stimulating hormone; HbA1C—glycated hemoglobin; Me—median; Q1—1st quartile; Q3—3rd quartile; N—group size; H—the test value in the Kruskal–Wallis test; η^2^—effects size (eta squared); *p* value—statistical significance: * in the overall Kruskal–Wallis test, in parentheses *p* values between groups from Dunn’s test with Bonferroni correction.

**Table 4:** Results of multivariate logistic regression analyses for selected emotional disorders.

Explained Variables	Predictors	OR	CI_low	CI_high	*p* Value	C-Index	Nagelkerke R^2^
anxiety or fear (I model)	(Intercept)	0.400	0.215	0.745	0.004	0.566	0.017
	gender (male)	0.680	0.537	0.862	0.001		
	age	1.049	0.998	1.102	0.062		
	excess body weight	0.890	0.652	1.214	0.461		
	underweight	0.973	0.553	1.715	0.926		
inadequate sadness (II model)	(Intercept)	0.131	0.065	0.264	<0.001	0.634	0.062
	gender (male)	0.471	0.361	0.616	<0.001		
	age	1.120	1.060	1.184	<0.001		
	excess body weight	0.892	0.631	1.262	0.520		
	underweight	0.627	0.307	1.278	0.199		
depression (III model)	(Intercept)	0.019	0.008	0.047	<0.001	0.683	0.094
	gender (male)	0.428	0.305	0.601	<0.001		
	age	1.231	1.147	1.320	<0.001		
	excess body weight	1.427	0.960	2.120	0.079		
	underweight	0.901	0.391	2.074	0.806		
excessive agitation (IV model)	(Intercept)	0.205	0.099	0.424	<0.001	0.560	0.013
	gender (male)	1.484	1.124	1.959	0.005		
	age	1.005	0.948	1.065	0.866		
	excess body weight	1.228	0.870	1.734	0.243		
	underweight	0.800	0.396	1.618	0.535		
chronic fatigue (V model)	(Intercept)	0.012	0.005	0.026	<0.001	0.691	0.117
	gender (male)	0.688	0.518	0.913	0.010		
	age	1.320	1.240	1.406	<0.001		
	excess body weight	0.982	0.683	1.412	0.921		
	underweight	0.489	0.213	1.121	0.091		
anger attacks (VI model)	(Intercept)	0.825	0.445	1.531	0.543	0.551	0.016
	gender (male)	1.272	1.004	1.612	0.046		
	age	0.961	0.914	1.010	0.114		
	excess body weight	0.947	0.696	1.288	0.728		
	underweight	0.564	0.303	1.050	0.071		

OR—odds ratio, CI—confidence intervals.

## Data Availability

The data presented in this study are available on request from the corresponding author due to privacy restrictions.

## Abbreviations

The following abbreviations are used in this manuscript:

BMI	body mass index
CIs	confidence intervals
COVID-19	coronavirus disease 2019
FG	fasting glucose
HbA1c	glycated hemoglobin
HBSC	Health Behaviour in School-aged Children
HDL-C	high-density lipoprotein cholesterol
IOTF	International Obesity Task Force
LDL-C	low-density lipoprotein cholesterol
Me	median
NHANES	National Health and Nutrition Examination Survey
non-HDL-C	non-high-density lipoprotein cholesterol
OR	odds ratio
PESEL	Universal Electronic System for Registration of the Population
PICTURE	Population Cohort Study of Wroclaw Citizens
PURE	Prospective Urban and Rural Epidemiological Study
SD	standard deviation
SDGs	Sustainable Development Goals
TGs	triglycerides
TSH	thyroid-stimulating hormone
Q1	1st quartile
Q3	3rd quartile
WHO	World Health Organization
